# Factors Predicting Short Length of Stay in Hospice Patients With a Primary Diagnosis of Stroke

**DOI:** 10.1093/geroni/igaa057.1348

**Published:** 2020-12-16

**Authors:** Victoria Marino, Maureen Templeman, Ronald Schonwetter, Debra Dobbs, William Haley

**Affiliations:** 1 University of South Florida, Tampa, Florida, United States; 2 Chapters Health System, Tampa, Florida, United States

## Abstract

Stroke is the second leading cause of death globally among people aged 60+, yet only 9% of hospice decedents have a primary diagnosis of stroke and little research has examined their end-of-life care experiences. Late referral and admission to hospice is an indicator of poor end of-life-care quality. This project identified factors predicting short stays in hospice, defined as a length of stay (LOS) of 14 days or less, via chart review of 100 hospice patients with a primary diagnosis of stroke. Of the 98 patients with complete data, 89% died in hospice; 11% were live discharges. Most patients were female, married, and referred to hospice from a hospital. Only 21% of patients entered hospice with a completed advance directive. Approximately 75% (n=73) of patients had a short LOS. Binary logistic regression indicated that gender, race, marital status, and having an advance directive at hospice admission were unassociated with LOS. Controlling for demographics, patients referred from home were 74% less likely to have a short LOS (OR=.26, CI=.08-.83) compared to those referred from a hospital, with a mean LOS of 44 and 13 days, respectively. Including Palliative Performance Scale (PPS) score in the model attenuated the effect of referral location. For every ten percent increase in PPS, participants were 85% less likely to have a short LOS (OR=.15, CI=.07-.32). Advance care planning should be more widely promoted among patients at high risk for stroke. There are opportunities for earlier referral to hospice for patients with a diagnosis of stroke.

